# Optimization of fermentation conditions for surfactin production by *B*. *subtilis* YPS-32

**DOI:** 10.1186/s12866-023-02838-5

**Published:** 2023-04-26

**Authors:** Yingjun Zhou, Xiaoxue Yang, Qing Li, Zheng Peng, Jianghua Li, Juan Zhang

**Affiliations:** 1 Science Center for Future Foods, Jiangnan University, Wuxi, 214122 China; 2 Key Laboratory of Industrial Biotechnology, School of Biotechnology, Ministry of Education, Jiangnan University, Wuxi, 214122 China; 3 Engineering Research Center of Ministry of Education on Food Synthetic Biotechnology, Jiangnan University, Wuxi, 214122 China; 4 Jiangsu Province Engineering Research Center of Food Synthetic Biotechnology, Jiangnan University, Wuxi, 214122 China; 5 Qingdao Vland Biotech Group Co., Ltd, Qingdao, 266000 China

**Keywords:** *B*. *subtilis* YPS-32, Surfactin, Plackett-Burman, Box-Behnken, Landy medium

## Abstract

**Background:**

Surfactin produced by microbial fermentation has attracted increasing attention because of its low toxicity and excellent antibacterial activity. However, its application is greatly limited by high production costs and low yield. Therefore, it is important to produce surfactin efficiently while reducing the cost. In this study, *B*. *subtilis* strain YPS-32 was used as a fermentative strain for the production of surfactin, and the medium and culture conditions for the fermentation of *B*. *subtilis* YPS-32 for surfactin production were optimized.

**Results:**

First, Landy 1 medium was screened as the basal medium for surfactin production by *B*. *subtilis* strain YPS-32. Then, using single-factor optimization, the optimal carbon source for surfactin production by *B*. *subtilis* YPS-32 strain was determined to be molasses, nitrogen sources were glutamic acid and soybean meal, and inorganic salts were KCl, K_2_HPO_4_, MgSO_4_, and Fe_2_(SO_4_)_3_. Subsequently, using Plackett-Burman design, MgSO_4_, time (h) and temperature (°C) were identified as the main effect factors. Finally, Box-Behnken design were performed on the main effect factors to obtain optimal fermentation conditions: temperature of 42.9 °C, time of 42.8 h, MgSO_4_ = 0.4 g·L^− 1^. This modified Landy medium was predicted to be an optimal fermentation medium: molasses 20 g·L^− 1^, glutamic acid 15 g·L^− 1^, soybean meal 4.5 g·L^− 1^, KCl 0.375 g·L^− 1^, K_2_HPO_4_ 0.5 g·L^− 1^, Fe_2_(SO_4_)_3_ 1.725 mg·L^− 1^, MgSO_4_ 0.4 g·L^− 1^. Using the modified Landy medium, the yield of surfactin reached 1.82 g·L^− 1^ at pH 5.0, 42.9 ℃, and 2% inoculum for 42.8 h, which was 2.27-fold higher than that of the Landy 1 medium in shake flask fermentation. Additionally, under these optimal process conditions, further fermentation was carried out at the 5 L fermenter level by foam reflux method, and at 42.8 h of fermentation, surfactin reached a maximum yield of 2.39 g·L^− 1^, which was 2.96-fold higher than that of the Landy 1 medium in 5 L fermenter.

**Conclusion:**

In this study, the fermentation process of surfactin production by *B*. *subtilis* YPS-32 was improved by using a combination of single-factor tests and response surface methodology for test optimization, which laid the foundation for its industrial development and application.

## Preface

Surfactin is a lipopeptide mainly produced by *Bacillus* [1]. It has good antibacterial, antiviral and biosurfactant activities, and antitumor effects [2, 3]. It has promising applications in food, agriculture, industry and medicine [4–8].

Currently, surfactin is mainly produced by microbial fermentation, but its low yield and high production cost severely limit its industrial production and application [9, 10]. The synthesis of surfactin is influenced by the composition of the medium such as carbon and nitrogen sources and culture conditions such as temperature, pH, and dissolved oxygen, and it is important to enhance the yield of surfactin while reducing the cost through scientific and rational optimization of fermentation conditions [11, 12]. Among them, the fermentation of inexpensive agricultural and industrial wastes as carbon and nitrogen sources for surfactin production is an effective way to reduce production costs. Currently, hydrolysed olive mill waste [13], cassava-processing effluent [14], waste distillers’ grains [15], and waste glycerol [16] have been used to effectively increase the production of *Bacillus* surfactin.

In this study, the surfactin-producing strain *B*. *subtilis* YPS-32 obtained by our group from previous screenings was used as a fermentative strain [17]. In this study, we performed initial screening of fermentation media for surfactin production by this *B. subtilis* YPS-32 strain. Then the inexpensive carbon and nitrogen sources for surfactin production by this bacterium were screened using a single-factor test and metal ion species were simplified. Finally, response surface methodology was used to optimize the important factors affecting the yield of surfactin, a medium suitable for the industrial production of surfactin fermentation was selected, and a 5 L fermenter was validated to lay the foundations for the industrial production of surfactin.

## Materials and methods

### Strain and culture conditions

The surfactin-producing strain in this study, *B*. *subtilis* YPS-32, was obtained in a previous study by atmospheric and room temperature plasma (ARTP) mutagenesis screening [17].

*B. subtilis* YPS-32 seed culture conditions: activated strains were streaked onto LB plates without antibiotics, and single colonies were picked and inoculated into LB liquid medium and cultured overnight at 37 ℃, 220 r·min^− 1^.

*B. subtilis* YPS-32 fermentation conditions: in a 250 mL shaker flask containing 50 mL of fermentation medium, the seed culture was inoculated at a ratio of 2%, placed at 30 ℃ and incubated at 220 r·min^− 1^ for 48 h.

### Rapid detection of surfactin content

According to a previous report, cetylpyridinium chloride-bromothymol blue (CPC-BTB) colorimetric assays were used for the rapid detection of surfactin [18]. Surfactin is a negatively charged lipopeptide biosurfactant that binds strongly to the cationic CPC, based on the principle that the colour indicator BTB first binds to the mediator CPC, resulting in a colour change from dark blue to pale yellow-green. The CPC is then competitively captured by surfactin from the CPC-BTB complex, releasing free molecules of BTB and producing a second colour change from a faint yellow-green to a dark green or bright blue [18]. Specifically, CPC-BTB solution was prepared by adding equal volumes of 0.2 mmol/L CPC and 0.2 mmol/L BTB in 0.1 mol/L PBS (phosphate buffered saline, NaH_2_PO_4_/Na_2_HPO_4_, pH 8.0). 100 µL of surfactin standard at different concentrations was added to 800 µL of CPC-BTB solution, and was allowed to react at 25 °C for 5 min. After reacting, 120 µL of the solution was transferred to a 96 well plate (transparent) and optical absorbance values at 600 nm were measured. A standard curve of surfactin was made according to the reading at *OD*_600_ nm by the CPC-BTB colorimetric assay and the concentration of the standard [18].

Sample pre-treatment: The fermentation broth was taken at the end of fermentation, centrifuged at 4 ℃ and 12,000 r·min^− 1^ for 10 min, the supernatant was filtered through a 0.22 μm aqueous membrane, and the surfactin content was determined using the above method.

### Quantification of surfactin content by HPLC

High Performance Liquid Chromatography (HPLC) is a good choice for the separation of target products, but since surfactin has many homologues, a suitable separation method is needed for both standards and samples to obtain good separation. The basic chromatographic conditions were an ODS-2 HypersilTM (250 × 4.6 mm) column with a detection wavelength of 210 nm, a column temperature of 35 ℃, and an injection volume of 10 µL. The separation methods were: 0–9 min: acetonitrile (0.1% Trifluoroacetic acid, TFA) 60–93%, water (0.1% TFA) 40 − 7%; 9–20 min: acetonitrile (0.1% TFA) 93%, water (0.1% TFA) 40 − 7%, water (0.1% TFA) 7%; total flow rate 0.84 mL·min^− 1^.

Standard curve establishment: surfactin standard was dissolved in ultrapure water to prepare a mother solution of 10 g·L^− 1^, and diluted to 0.2 g·L^− 1^, 0.5 g·L^− 1^, 1 g·L^− 1^, 2.5 g·L^− 1^, 5 g·L^− 1^, 10 g·L^− 1^ using ultrapure water. The surfactin content was determined using the liquid phase method described above, and a standard curve was established based on the sum of the peak areas and the concentrations of the standards. The sample pre-treatment method was the same as that for CPC-BTB colorimetric assays.

### Initial screening of fermentation media

Four commonly used bacterial culture media (NB, LB, BPY, and NYD) and three *Bacillus subtilis* surfactin-producing media (Landy, Landy 1, Landy 2) reported in the literature were selected (Table 1). The seed liquid of *B*. *subtilis* YPS-32 was inoculated at a ratio of 2% and left to ferment at 30 °C, 220 r·min^− 1^ for 48 h. After fermentation, surfactin yields were measured using the CPC-BTB colorimetric assay, and the optimum medium for surfactin production by *B*. *subtilis* YPS-32 was selected and used as the basis for the optimization of subsequent culture conditions.

**Table 1:** Culture medium and its composition

Medium	Composition (g/L)
NB medium	Beef extract 5, peptone 10, NaCl 5
LB medium	Yeast powder 5, peptone 10, NaCl 5
BPY medium	Beef extract 5, peptone 10, yeast powder 5, NaCl 5, glucose 10
NYD medium	Beef extract 8, yeast powder 3, glucose 1
Landy medium [19]	Glucose 20, L-glutamic acid 5, MgSO_4_ 0.5, KCl 0.5, KH_2_PO_4_ 1, FeSO_4_ 0.00015, MnSO_4_ 0.0005, CuSO_4_ 0.00016
Modified Landy medium 1 (Landy 1) [20]	Glucose 20, L-glutamic acid 5, yeast powder 1, K_2_HPO_4_ 1, MgSO_4_ -7H_2_ O 1.02, KCl 0.5, CuSO_4_ -5H_2_ O 0.0025, Fe_2_(SO_4_)_3_ 0.0004, MnSO_4_·H_2_O 0.00134
Modified Landy medium 2 (Landy 2) [21]	Glucose 19.97, L-glutamic acid 13.51, yeast powder 1, K_2_HPO_4_ 1, MnSO_4_·7H_2_O 0.5, KCl 0.5, L-tryptophan 0.06312, CuSO_4_·5H_2_O 0.0016, MnSO_4_·H_2_O 0.0012, Fe_2_(SO_4_)_3_·7H_2_O 0.0004

### Effect of carbon source on surfactin yield

On the basis of Landy 1 medium, the carbon source in the medium was screened using 20 g/L molasses, starch, sucrose, fructose, galactose, and glycerol in the replacement of 20 g/L glucose, while other components remained unchanged. After fermentation, the surfactin content was detected using the CPC-BTB colorimetric assay.

### Effect of nitrogen source on surfactin yield

On the basis of Landy 1 medium, the nitrogen source in the medium was screened using 1 g/L soybean meal, bran, wheat flour, corn pulp, ammonium sulphate and ammonium nitrate in the replacement of 1 g/L yeast powder, without adjustment of the other components. After fermentation, the surfactin content was detected using the CPC-BTB colorimetric assay.

### Forward single-factor experiment involving metal ions

Using glucose, glutamic acid and yeast powder as the base medium, KCl, K_2_HPO_4_, MgSO_4_·7H_2_O, Fe_2_(SO_4_)_3_, MnSO_4_·H_2_O, CuSO_4_·5H_2_O were each added as the original medium for the experimental group, and their the final concentrations were 0.5, 1, 1.02, 0.0004, 0.00134, 0.0025 g/L, respectively. After fermentation, the surfactin content was detected using the CPC-BTB colorimetric assay, and statistical analysis was performed with IBM SPSS Statistics software using independent sample Student’s *t*-tests.

### Reverse single-factor experiments involving metal ions

On the basis of Landy1 medium, with glucose, glutamic acid, and yeast powder left unchanged, KCl, K_2_HPO_4_, MgSO_4·_7H_2_O, Fe_2_(SO_4_)_3_, MnSO_4·_H_2_O, CuSO_4·_5H_2_O were each subtracted as the medium for the experimental group. After fermentation, the surfactin content was detected using the CPC-BTB colorimetric assay, and the results were analysed using IBM SPSS software for statistical analysis using independent sample Student’s *t*-tests.

### Plackett-Burman design

Based on the determination of carbon source, nitrogen source, and metal ions in the medium, the Plackett-Burman design (PBD) was used to determine the main effect factors of the medium components: carbon source (molasses), nitrogen source (soybean meal, glutamic acid), metal ions (KCl, K_2_HPO_4_, MgSO_4_, Fe_2_(SO_4_)_3_) and fermentation conditions (fermentation time, pH, fermentation temperature, inoculum size). At the end of fermentation, surfactin content was measured using the CPC-BTB colorimetric assay with surfactin content as the experimental response value. Minitab software was used to perform the PBD for n = 11, and the factors and levels of the PBD are shown in Table 2.

**Table 2:** Plackett-Burman design factors and levels

Factor	Variable	Level	
		Low level (-1)	High level (+ 1)
X_1_	Molasses (g·L^− 1^)	20	30
X_2_	Glutamic acid (g·L^− 1^)	10	15
X_3_	Soybean meal (g·L^− 1^)	3	4.5
X_4_	KCl (g·L^− 1^)	0.25	0.375
X_5_	K_2_HPO_4_ (g·L^− 1^)	0.5	0.75
X_6_	MgSO_4_ (g·L^− 1^)	0.25	0.375
X_7_	Fe_2_(SO_4_)_3_ (mg·L^− 1^)	1.2	2.25
X_8_	Time (h)	30	45
X_9_	pH	5	7.5
X_10_	Temperature (℃)	28	35
X_11_	Inoculation size (%)	2	3

### Box-Behnken design based on response surface analysis

Response surface analysis is a statistical mathematical method used to reflect the best corresponding conditions obtained when interactions among factors in a multifactorial system are made to reach the maximal response value [22]. In this experiment, using Design-Expert software, the Box-Behnken design (BBD) was used to further evaluate the screened principal component factors (temperature, time, and MgSO_4_). The levels of the principal component factors were independent variables, and the low, medium and high experimental levels of each variable were − 1, 0 and 1, respectively. The CPC-BTB colorimetric assay was used to detect surfactin content with surfactin content as the experimental response. The factors and levels in the experimental protocol are shown in Table 3.

**Table 3:** Response surface experimental factors and levels

Code	Factor	Level		
		-1	0	1
A	Temperature (℃)	37	40	43
B	Time (h)	39	45	51
C	MgSO_4_ (g·L^− 1^)	0.3	0.35	0.4

### Model validation

Fermentation was carried out using the optimal fermentation conditions analysed by response surface analysis, and the surfactin content in the fermentation supernatants was determined using HPLC after the end of fermentation.

### Fermentation at the 5 L fermenter level

In this study, the design of Yeh et al. was used to modify the fermenter by adding a foam reflux part with reference to the design [23]. In this study, a 5 L fermenter was used to produce surfactin by fermentation of *B*. *subtilis* YPS-32 using the optimized fermentation conditions and the modified apparatus. With the loading volume at 2.5 L, the secondary seeds were inoculated into the fermenter at 2% inoculum, and the pH of the fermentation broth was maintained at 7.0 using 50% phosphoric acid. The fermentation aeration rate was 1 L·min^− 1^, and the rotation speed was 300 r·min^− 1^ in the early stage and the parameters were adjusted up and down according to foam production in the later stage. A 2 L sterile reflux flask was used to control the rate of foam reflux and the rate of foam flowing into the reflux flask to keep the volume of the fermentation broth at 2.5 L. Biomass and surfactin content were measured by sampling regularly during the fermentation process.

## Results

### Rapid detection method for surfactin content

**Fig. 1:**
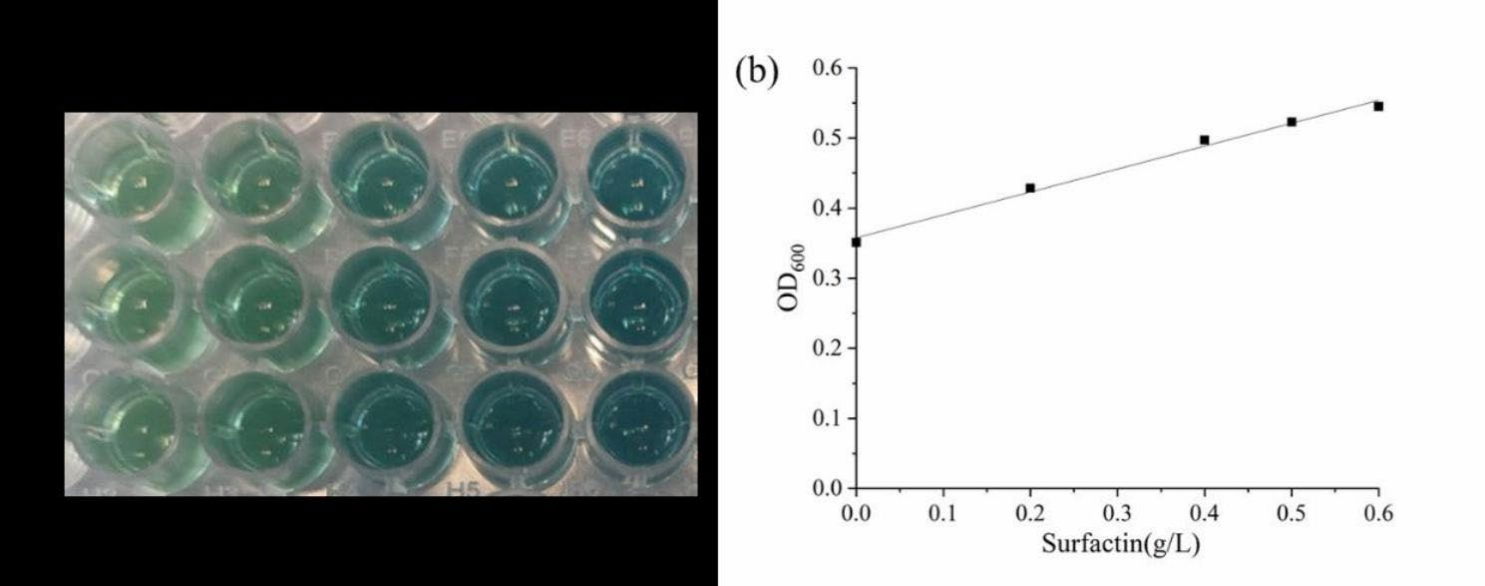
Detection of surfactin content by the CPC-BTB colorimetric assay (a) Example plate of the CPC-BTB colorimetric assay; (b) Standard curve detection by the CPC-BTB colorimetric assay for surfactin.

Rapid determination of surfactin content was performed using the CPC-BTB colorimetric assay, and the effects of different concentrations of surfactin are shown in Fig. 1a. A standard curve of surfactin was generated according to the reading at *OD*_600_ by the CPC-BTB colorimetric assay and the concentration of the standard is shown in Fig. 1b, with the equation of the standard curve being y = 0.327x + 0.3575 with a correlation coefficient R^2^ of 0.991, indicating a good correlation. Therefore, the CPC-BTB colorimetric assay can be used for the rapid determination of surfactin content in subsequent fermentation optimization processes.

### Identification of the method for the liquid-phase determination of surfactin content

**Fig. 2:**
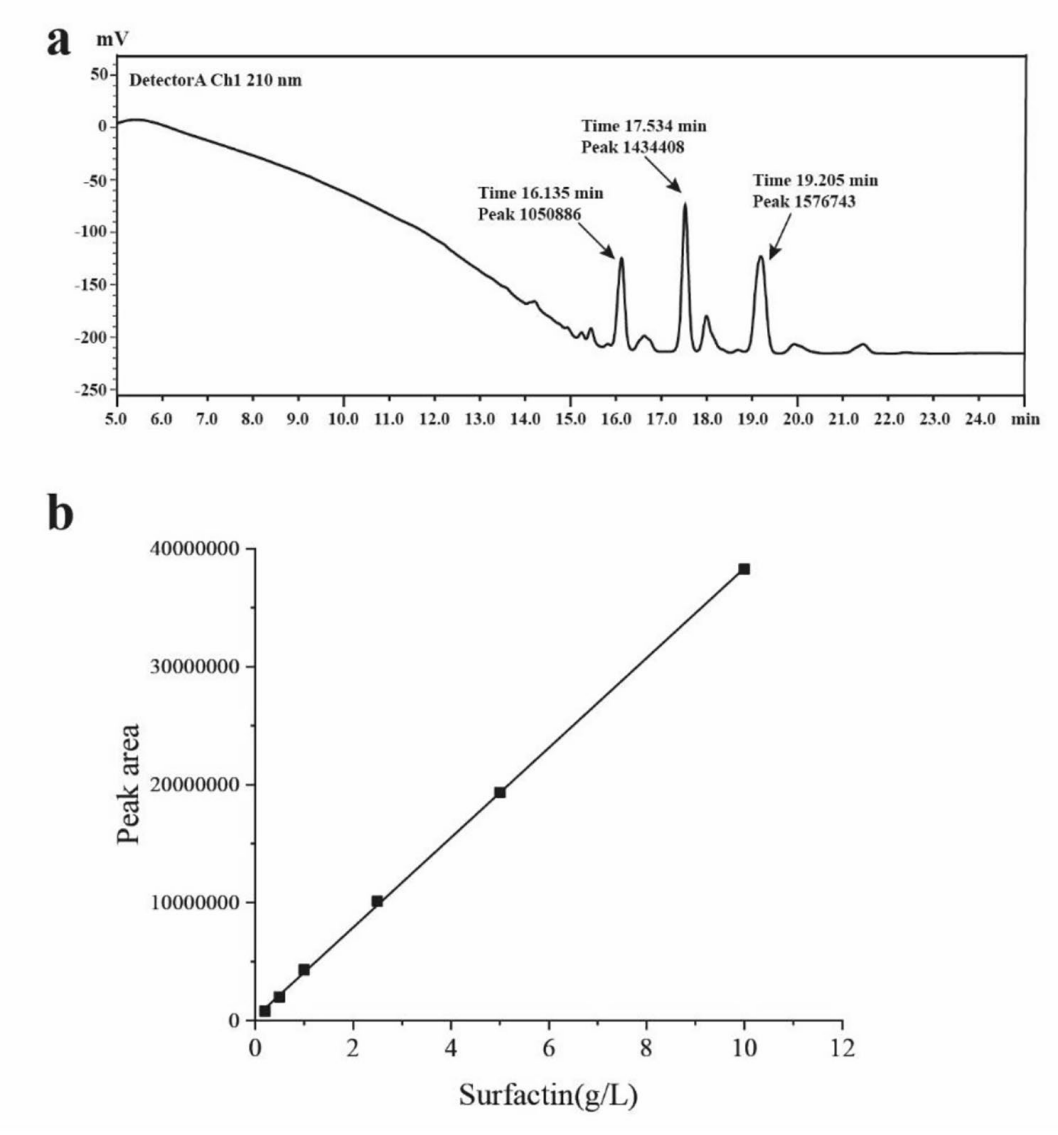
Detection of surfactin content by HPLC (a) Chromatograms of surfactin standard by HPLC; (b) Standard curve of surfactin determined by HPLC.

HPLC was used to analyse the peaks of the samples as shown in Fig. 2a. This method produced a smooth baseline peak and good separation of surfactin. The standard curve of surfactin was made according to the peak area of the liquid phase and the concentration of the standard as shown in Fig. 2b. The equation of the standard curve was y = 3,805,784,50x + 280,003.58, and the correlation coefficient R^2^ was 0.9997, demonstrating a good correlation.

### Screening of the initial fermentation medium

**Fig. 3:**
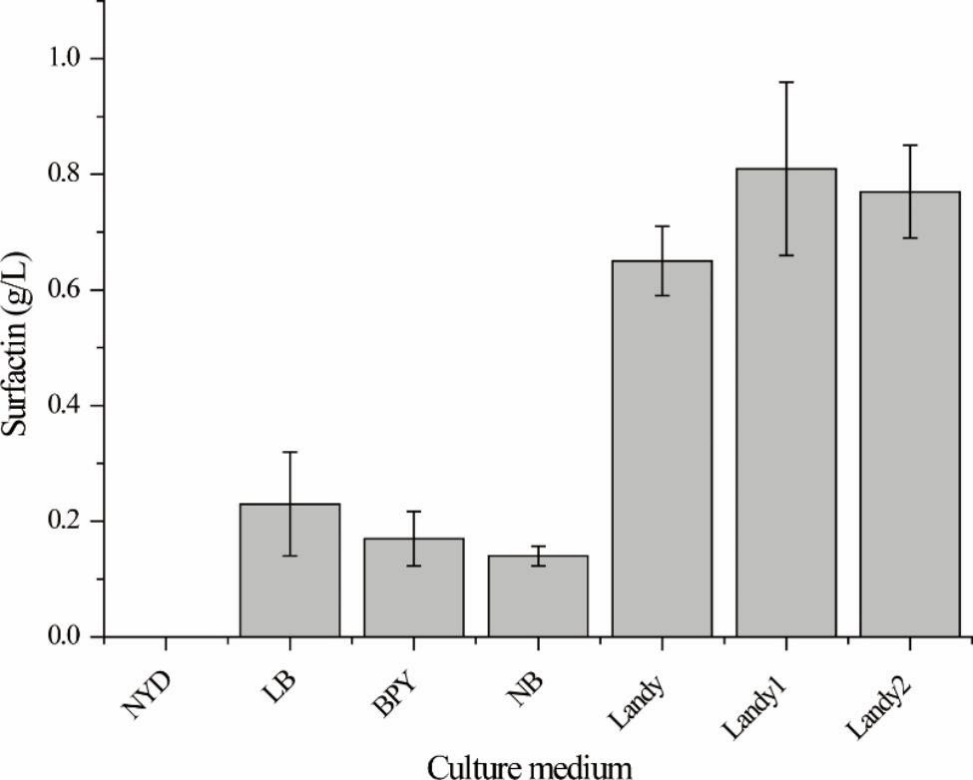
Effect of culture medium on surfactin content

Seven media were selected for the fermentation of *B. subtilis* strain YPS-32 under the same conditions, and the content of surfactin in the fermentation broth was detected according to the CPC-BTB colorimetric assay; the results are shown in Fig. 3. Surfactin was produced in all except NYD medium, and the surfactin content varied greatly between the different mediums. With the highest content of surfactin, Landy 1 medium was determined to be the basic medium for surfactin production by *B*. *subtilis* strain YPS-32.

### Determination of optimal carbon source

**Fig. 4:**
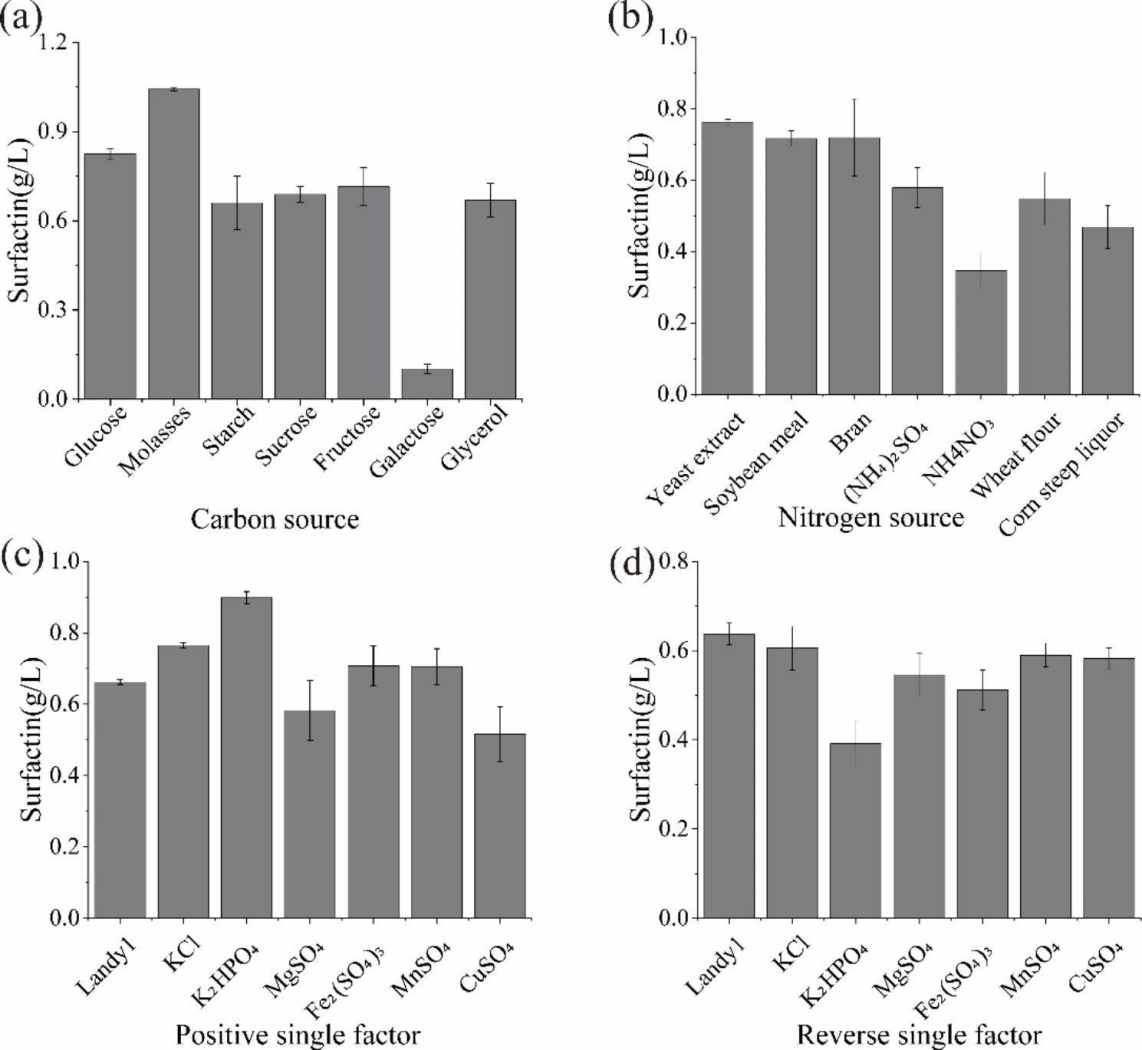
Results of single-factor tests (a) Effect of carbon source on surfactin content; (b) Effect of nitrogen source on surfactin content; (c) Results of metal ion forward single-factor experiments; (d) Results of metal ion reverse single-factor experiments.

The effects of molasses, starch, sucrose, fructose, galactose, glycerol, and control glucose on surfactin production by *B. subtilis* strain YPS-32 were investigated, and the results are shown in Fig. 4a. From the Figure, it can be seen that all carbon sources except galactose were beneficial in terms of surfactin production, and the highest surfactin production was achieved when molasses was the sole carbon source. The raw materials of molasses are easy to obtain and inexpensive, therefore, molasses was identified as the carbon source of the culture medium for subsequent optimization.

### Determination of optimal nitrogen source

Keeping the inexpensive nitrogen source glutamic acid unchanged, the effects of soybean meal, bran, wheat flour, corn pulp, ammonium sulphate, ammonium nitrate and control yeast powder on surfactin production by *B*. *subtilis* strain YPS-32 were investigated experimentally, and the results are shown in Fig. 4b. As can be seen from the Figure, surfactin production was slightly lower when using soybean meal or bran for fermentation than when yeast powder was used, but both are much less expensive than yeast powder. The price of soybean meal was lower than that of bran and the surfactin yield of both fermentation broths was comparable, so it was determined that soybean meal was to be used instead of yeast powder in the original medium for subsequent optimization.

### Determination of optimum metal ion

Many previous optimizations of Landy medium only optimized the amount of each component of the medium, but the composition of the medium did not change, which could not overcome the many disadvantages of Landy medium – too many components, cumbersome preparation, easy formation of precipitates involving different salts, etc. In this experiment, a two-way single-factor experimental method was used to further optimize the Landy medium to simplify the composition of the medium and reduce costs. Among them, the forward single-factor results are shown in Fig. 4c, and the reverse single-factor results are shown in Fig. 4d.

In the results of the forward single-factor experiment, the yield of surfactin in the experimental group with the addition of KCl was greater than that of the control, and the difference between the experimental group and the control group was significant by Student’s *t*-test (p = 0 < 0.05), indicating that KCl is beneficial to the synthesis of surfactin. From results of the reverse single-factor experiment, the yield of surfactin in the experimental group without the addition of KCl was slightly lower than that of the control, but the difference was not significant (p = 0.373 > 0.05). Although the results of the forward and reverse single-factor experiments were inconsistent, the results of the forward single-factor experiment indicated that KCl is beneficial to the synthesis of surfactin, while the results of the reverse single-factor experiment indicated that KCl had no adverse effect on the synthesis of surfactin, so the addition of KCl was chosen based on the results of the forward single-factor experiment.

From the results of the forward single-factor experiment, the yield of surfactin in the experimental group supplemented with K_2_HPO_4_ was greater than the control, and the difference was significant by Student’s *t*-test (p = 0 < 0.05), indicating that K_2_HPO_4_ was beneficial for surfactin synthesis. From the results of the reverse single-factor experiment, the yield of surfactin in the experimental group without the addition of K_2_HPO_4_ was less than the control, and the difference was significant by Student’s *t*-test (p = 0.002 < 0.05), therefore K_2_HPO_4_ is beneficial for surfactin synthesis, so the addition of K_2_HPO_4_ was chosen.

From the results of the forward single-factor experiment, the surfactin yield of the experimental group with the addition of MgSO_4·_7H_2_O was lower than the control, and the difference was not significant by Student’s *t*-test (p = 0.176 > 0.05), so MgSO_4·_7H_2_O had no significant effect on surfactin synthesis. From the results of the reverse single-factor experiment, in the experimental group without the addition of MgSO_4·_7H_2_O, surfactin yield was less than the control, and the difference was significant by Student’s *t*-test (p = 0.042 < 0.05), hence MgSO_4·_7H_2_O was beneficial for surfactin synthesis, and therefore the addition of MgSO_4·_7H_2_O was chosen.

From the results of the forward single-factor experiment, the yield of surfactin in the experimental group with the addition of Fe_2_(SO_4_)_3_ was slightly greater than that of the control, but the difference was not significant (p = 0.294 > 0.05), therefore Fe_2_(SO_4_)_3_ had little effect on surfactin synthesis. From the results of the reverse single-factor experiment, the yield of surfactin in the experimental group without the addition of Fe_2_(SO_4_)_3_ was less than that of the control, and the difference was significant (p = 0.013 < 0.05), indicating that Fe_2_(SO_4_)_3_ had a beneficial effect on surfactin synthesis. The difference was significant by Student’s *t*-test (p = 0.013 < 0.05), indicating that Fe_2_(SO_4_)_3_ was beneficial for surfactin synthesis. Although the results of the forward and reverse single-factor experiments were inconsistent, the results of the reverse single-factor experiment indicated that Fe_2_(SO_4_)_3_ is beneficial to the synthesis of surfactin, while the results of the forward single-factor experiment indicated that Fe_2_(SO_4_)_3_ had no adverse effect on the synthesis of surfactin, so the addition of Fe_2_(SO_4_)_3_ was chosen.

From the results of the forward single-factor experiment, the surfactin yield of the experimental group with the addition of MnSO_4·_H_2_O was slightly greater than that of the control, and the difference between the experimental group and the control group was not significant by Student’s *t*-test (p = 0.214 > 0.05), indicating that MnSO_4·_H_2_O had little effect on surfactin synthesis. From the results of the reverse single-factor experiment, the surfactin yield in the experimental group without the addition of MnSO_4·_H_2_O was slightly lower than the control, and this difference was not significant by Student’s *t*-test (p = 0.086 > 0.05), indicating that MnSO_4·_H_2_O had little effect on surfactin synthesis. Therefore, from the perspective of simplifying the medium formulation and reducing costs, MnSO_4·_H_2_O was chosen not to be added.

From the results of the forward single-factor experiment, the yield of surfactin in the experimental group with the addition of CuSO_4·_5H_2_O was slightly less than that of the control, and the difference was significant by Student’s *t*-test (p = 0.03 < 0.05), so CuSO_4·_5H_2_O had an unfavourable effect on surfactin synthesis. From the results of the reverse single-factor experiment, the yield of surfactin in the experimental group without the addition of CuSO_4·_5H_2_O was less than that of the control, and the Student’s *t*-test p = 0.049 was closer to 0.05, therefore CuSO_4·_5H_2_O had little effect on surfactin synthesis. Combining the results of both forward and reverse single-factor experiments, CuSO_4·_5H_2_O was chosen not to be added.

In summary, according to the results of the forward and reverse single-factor experiments, keeping glucose, glutamic acid and yeast powder unchanged on the basis of Landy 1 medium, KCl, K_2_HPO_4_, MgSO_4_ and Fe_2_(SO_4_)_3_ were selected to be added.

### Plackett-Burman design to screen the main effect factors on surfactin yield

The PBD was performed using Minitab software to select the main effect factors with significant effect on surfactin production, and the experimental results are shown in Table 4.

**Table 4:** Results of Plackett-Burman design

Experiment No.	X_1_	X_2_	X_3_	X_4_	X_5_	X_6_	X_7_	X_8_	X_9_	X_10_	X_11_	Surfactin (g/L)
1	-1	-1	1	1	1	-1	1	1	-1	1	-1	1.3750
2	-1	1	-1	-1	-1	1	1	1	-1	1	1	1.3944
3	-1	-1	-1	1	1	1	-1	1	1	-1	1	1.1375
4	-1	1	1	1	-1	1	1	-1	1	-1	-1	1.0509
5	1	-1	1	-1	-1	-1	1	1	1	-1	1	0.9245
6	1	-1	-1	-1	1	1	1	-1	1	1	-1	1.1120
7	1	1	-1	1	1	-1	1	-1	-1	-1	1	0.8307
8	1	1	1	-1	1	1	-1	1	-1	-1	-1	1.1232
9	1	-1	1	1	-1	1	-1	-1	-1	1	1	1.2700
10	1	1	-1	1	-1	-1	-1	1	1	1	-1	1.3465
11	-1	-1	-1	-1	-1	-1	-1	-1	-1	-1	-1	0.7899
12	-1	1	1	-1	1	-1	-1	-1	1	1	1	0.9520

Analysis of the results of the experimental design is shown in Table 5. We selected MgSO_4_, time (h), and temperature (°C) as the three most influential factors based on the magnitude of the p values, and p values being < 0.5, and these three factors are therefore significant factors that affect the production of surfactin by the strain. And according to Student’s *t*-value, MgSO_4_, time (h), and temperature (°C) were positively correlated with surfactin production and should be increased, while the remaining non-significant factors showing negative effects were taken at low level (-1) and positive effects were taken at high level (+ 1) for the experiment, i.e. molasses 20 g·L^− 1^, glutamic acid 15 g·L^− 1^, soybean meal 4.5 g·L^− 1^, KCl 0.375 g·L^− 1^, and K_2_HPO_4_ 0.5 g·L^− 1^ at pH 5.0 and 2% inoculum.

**Table 5:** Estimated effects and coefficients of the response values of PBD experiment

Item	Effect	Coefficient	Standard error of coefficient	*t*-value	p-value
Constant		0.36931	0.00186	198.43	0.003
Molasses (g·L^− 1^)	-0.00506	-0.00253	0.00186	-1.36	0.404
Glutamic acid (g·L^− 1^)	0.00483	0.00242	0.00186	1.3	0.418
Soybean meal (g·L^− 1^)	0.00461	0.00231	0.00186	1.24	0.432
KCl (g·L^− 1^)	0.03894	0.01947	0.00186	10.46	0.061
K_2_HPO_4_ (g·L^− 1^)	-0.01339	-0.00669	0.00186	-3.6	0.173
MgSO_4_ (g·L^− 1^)	0.04739	0.02369	0.00186	12.73	0.05
Time (h)	0.07061	0.03531	0.00186	18.97	0.034
pH	-0.01417	-0.00708	0.00186	-3.81	0.164
Temperature (℃)	0.08683	0.04342	0.00186	23.33	0.027
Inoculum size (%)	-0.01572	-0.00786	0.00186	-4.22	0.148

### Box-Behnken design and result analysis

Design-Expert software was used for BBD and results analysis, with MgSO4, time (h), and temperature (℃) as experimental factors, surfactin yield as the response value, and three-factor, three-level optimization experiments were performed, and the specific experimental design and results are shown in Table 6. The regression equation was obtained as: Y = 1.4357 + 0.2431 A + 0.0757B – 0.0096 C – 0.1422AB + 0.1177AC + 0.0457BC – 0.0339A^2^ – 0.079766B^2^ + 0.0094C^2^.

Where Y is the surfactin yield (g/L), A, B, and C are temperature, time, and MgSO_4_, respectively.

**Table 6:** Box-Behenken design experiment

Run sequence	A	B	C	Y (g/L)
1	-1	-1	0	0.9260
2	0	-1	-1	1.2557
3	1	-1	0	1.6936
4	0	0	0	1.4550
5	1	0	1	1.7379
6	0	0	0	1.4795
7	1	0	-1	1.5737
8	-1	0	-1	1.3199
9	0	1	1	1.5667
10	0	0	0	1.3725
11	-1	1	0	1.2349
12	-1	0	1	1.0132
13	1	1	0	1.4336
14	0	-1	1	1.1966
15	0	1	-1	1.4422

The regression model was analysed and the results are shown in Table 7. Item A had a highly significant effect on Y values (p < 0.001), term AB had a significant effect on Y values (p < 0.05), and the remaining terms did not have significant effects. The model p = 0.0139 < 0.05, indicating that the regression is significant, and the misfit term p = 0.2069 > 0.05, indicating that the misfit is not significant, and the model R^2^ = 0.9403, indicating that 94.03% of the experimental results can be explained using the model, and R^2^ adj = 0.8328, indicating that the actual value is also close to the predicted value of the model, therefore, the combination indicates that the model has high credibility that can be used to predict changes in surfactin yields.

**Table 7:** Box-Behenken design experimental results

Source	Sum of Squares	df	Mean Square	F Value	P-value Prob > F	
Model	0.69	9	0.077	8.75	0.0139	significant
A-Temperature	0.47	1	0.47	53.82	0.0007	
B-Time	0.046	1	0.046	5.22	0.0712	
C-MgSO_4_	7.424E-004	1	7.424E-004	0.084	0.7830	
AB	0.081	1	0.081	9.21	0.0289	
AC	0.055	1	0.055	6.31	0.0537	
BC	8.417E-003	1	8.417E-003	0.96	0.3726	
A^2^	4.242E-003	1	4.242E-003	0.48	0.5181	
B^2^	0.023	1	0.023	2.67	0.1629	
C^2^	3.247E-004	1	3.247E-004	0.037	0.8551	
Residual	0.044	5	8.786E-003			
Lack of Fit	0.038	3	0.013	3.99	0.2069	Not significant
Pure Error	6.291E-003	2	3.145E-003			
Cor Total	0.74	14			0.0139	

**Fig. 5:**
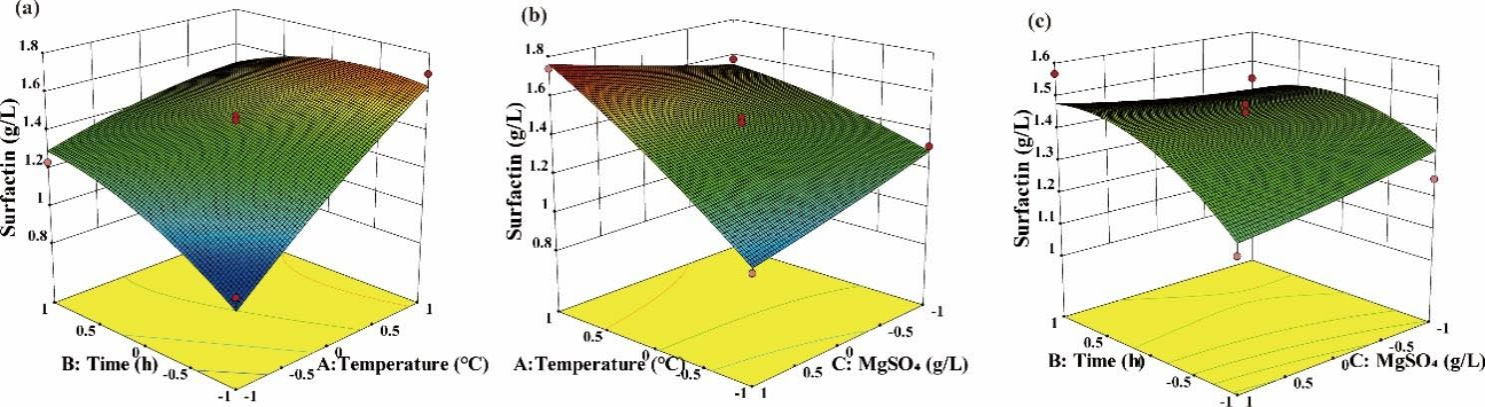
Response surface plots of MgSO_4_, time (h), and temperature (°C) on surfactin yields (a) Response surface plot of temperature and time; (b) Response surface plot of temperature and MgSO_4_; (c) Response surface plot of time and MgSO4.

The three-dimensional response surface of surfactin production was plotted according to the regression model to visualize effects of the independent variables on response values, and by keeping the third variable at the zero level to compare the magnitude of the effects of the other two variables on response values. The greater the degree of slope curvature in the response surface, the greater the effect of the factors on the response value. According to the response surface analysis plots, it can be seen that in the interaction plot between fermentation time and temperature (Fig. 5a), the degree of curvature of fermentation temperature is greater extent than that of time, indicating that the effect of temperature on surfactin yield is greater than time. In the interaction plot between fermentation temperature and medium MgSO_4_ addition (Fig. 5b), the degree of curvature of fermentation temperature is greater than that for MgSO_4_ addition, indicating that temperature has a greater effect on surfactin yield than MgSO_4_ addition. In the interaction plot between fermentation time and medium MgSO_4_ addition (Fig. 5c), the degree of curvature of fermentation time is more than that of MgSO_4_ addition, indicating that time has a greater impact on the yield of surfactin than MgSO_4_ addition.

According to the response surface analysis plot, it can be seen that surfactin yield has a theoretical maximum of 1.74 g·L^− 1^ at 42.9 ºC, 42.8 h, and MgSO_4_ = 0.4 g·L^− 1^.

### Model validation

The use of the CPC-BTB colorimetric assay enabled rapid optimization of the fermentation process for surfactin, and HPLC was then used to verify surfactin content due to the high accuracy and sensitive detection of the substance. The optimal fermentation conditions arrived at using response surface analysis were used for fermentation validation experiments under these optimal conditions, i.e. molasses 20 g·L^− 1^, glutamic acid 15 g·L^− 1^, soybean meal 4.5 g·L^− 1^, KCl 0.375 g·L^− 1^, K_2_HPO_4_ 0.5 g·L^− 1^, Fe_2_(SO_4_)_3_ 1.725 mg·L^− 1^, MgSO_4_ 0.4 g·L^− 1^, pH 5.0, inoculum size 2%, incubation temperature 42.9℃, incubation time 42.8 h. The concentration of surfactin in the fermentation supernatant was determined by HPLC at the end of fermentation to be 1.82 g·L^− 1^. The actual measured yield was close to the predicted yield, indicating that the established model was in agreement with the actual situation, and therefore optimization of the fermentation conditions of the strain for maximum surfactin by response surface methodology was effective and feasible.

### Scaled-up experiments in 5 L fermenter

**Fig. 6:**
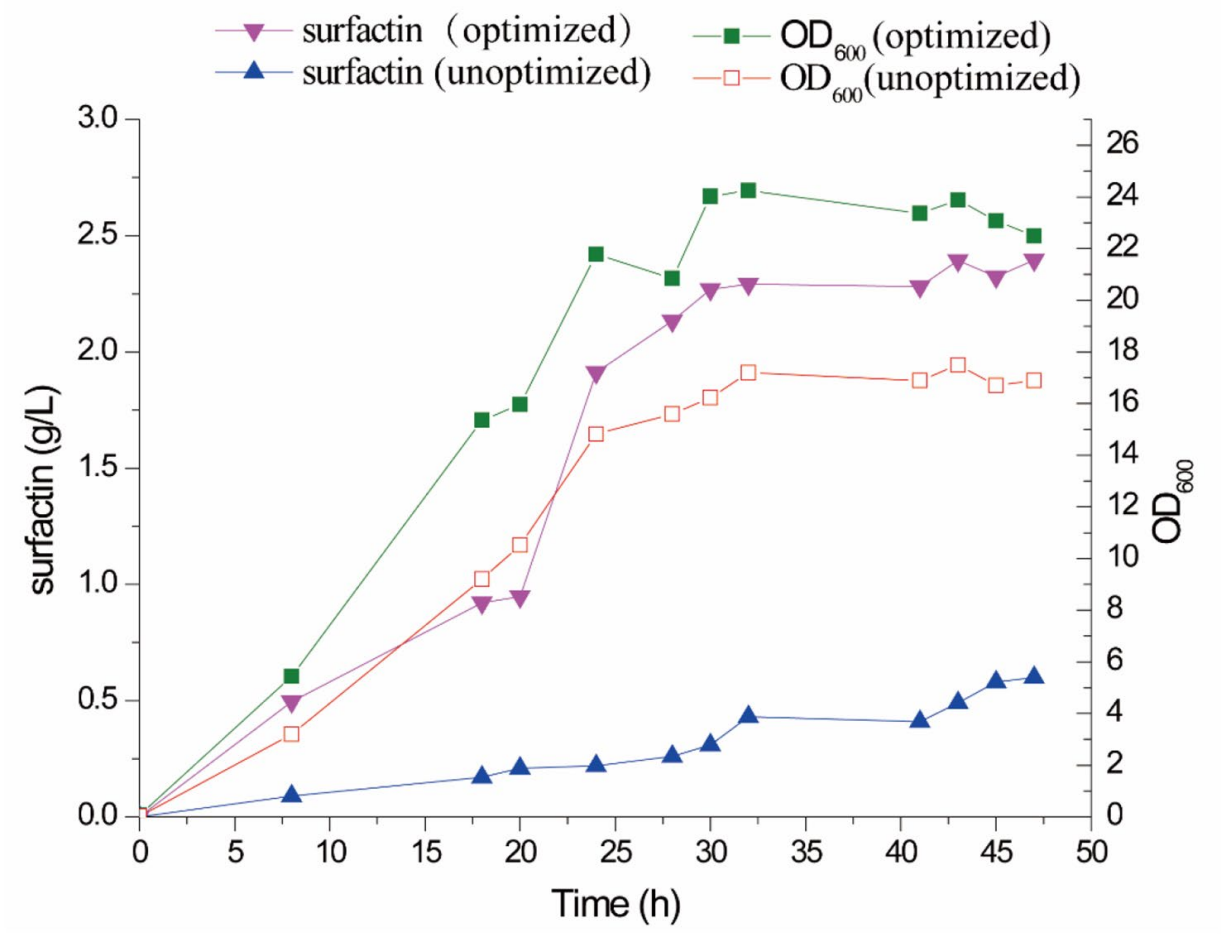
Surfactin production curve of *B. subtilis* strain YPS-32 in 5 L fermenter

The growth curve of the bacterium and the product synthesis process at the 5 L fermenter level under optimized and unoptimized medium and culture conditions were examined and the results are shown in Fig. 6. The results showed that the synthesis of surfactin was coupled with the growth of the bacterium and the product was synthesized continuously with the growth of the bacterium. After 42.8 h of fermentation, there was a maximum yield of surfactin of 2.39 g·L^− 1^, which was 2.96-fold higher than that of the Landy 1 medium in 5 L fermenter. Compared with fermentation levels in shaker flasks, the biomass at the end of fermentation in the 5 L fermenter was twice as high as that in the shaker flask, indicating that the foam reflux method allows the nutrients in the culture medium to be fully utilized. Taken together, the results showed that growth and surfactin production by the bacteria were significantly enhanced at the 5 L fermenter level compared with shaker flask fermentation, probably because the fermenter system had more suitable pH and dissolved oxygen conditions than the shaker flask fermentation method, and the foam reflux method also effectively prevented overflow of medium.

## Discussion

The determination of surfactin content by HPLC is accurate and sensitive, whereas the detection cost is high and time-consuming. According to the literature, CPC-BTB colorimetric assays are used for the rapid determination of surfactin content with simple detection reagents and easy achievement of high throughput detection, which can greatly accelerate the optimization process compared with the traditional HPLC method for determination of surfactin content. Therefore, in this study, we used the CPC-BTB colorimetric assay for the rapid determination of surfactin content in the fermentation optimization process.

Previous studies have shown that inexpensive industrial and agricultural by-products can be used as potential resources for surfactin production [24]. The use of by-products as carbon and nitrogen sources for surfactin production is an effective means to reduce production costs. In this study, the effect of carbon sources (molasses, starch, sucrose, fructose, galactose, glycerol, and glucose) on surfactin production by *B*. *subtilis* strain YPS-32 was investigated and the results showed that molasses were the best carbon source for surfactin production. This result is in agreement with the findings of Abdel-Mawgoud et al. in that *B*. *subtilis* BS5 also had the highest surfactin production when fermenting with molasses as the sole carbon source, which could successfully replace glucose [25]. In addition, the effects of nitrogen sources (soybean meal, bran, wheat flour, corn pulp, ammonium sulphate, ammonium nitrate, and yeast powder) on the yield of surfactin were investigated. The results showed that surfactin yield was slightly lower when using soybean meal or bran for fermentation than when using yeast powder, but both were much less expensive than yeast powder. In addition, the price of soybean meal was lower than that of bran and the surfactin yield of both fermentation broths was comparable, so it was determined that soybean meal be used as the nitrogen source for surfactin production. This result is consistent with the findings of Zhu et al. who examined the effects of using canola meal, corn meal, soybean meal, bran, soybean cake powder and rice husk powder as substrates on surfactin production using *Bacillus amyloliquefaciens* XZ-173 as the fermentation strain. The results also showed that the highest surfactin yield was achieved when soybean meal was used as substrate [26]. Therefore, the results of this study suggest that surfactin can be produced by fermentation using inexpensive molasses and soybean meal as carbon and nitrogen sources, respectively, which would significantly reduce the cost of surfactin production.

Response surface analysis is an effective method for optimizing fermentation parameters [27]. For example, Zhu et al. obtained optimal fermentation parameters for surfactin production by fermentation using *Bacillus amyloliquefaciens* strain XZ-173 through PBD and BBD. In this study, the optimal carbon, nitrogen, and metal ion sources for surfactin production were determined using single-factor tests, and a PBD was used to screen the main effect factors with significant effects on surfactin production. Response surface analysis was then used to analyse the fermentation parameters that achieved the highest surfactin production. Under optimal fermentation conditions, the yield of surfactin reached 1.82 g·L^− 1^, which was similar to the predicted value, indicating that it is practical to optimize the fermentation conditions for surfactin production using response surface methodology.

Surfactin has good surface activity and is susceptible to foam formation during fermentation due to agitation and aeration, which has become an important problem in industrial production. However, the addition of defoamer may adversely affect cell growth and metabolism, and its high cost and difficulty in separation from the product limit its application [28]. Foam separation technology can effectively solve the problem of difficult foam control during fermentation without adding any defoamer, which reduces production costs and is easy to produce on a large scale with simple equipment and low energy consumption [29]. Therefore, in this study, we used foam separation technology for surfactin fermentation in a 5 L fermenter, and growth and surfactin production capacity of the bacteria were significantly increased at the 5 L fermenter level.

## Conclusions

In this study, Landy 1 medium was firstly determined to be used as the basal medium for surfactin production by *B. subtilis* strain YPS-32. Then, the fermentation process for surfactin production by *B*. *subtilis* strain YPS-32 was optimized using a combination of single-factor and response surface approaches. The yield of surfactin under shaker flask fermentation conditions reached 1.82 g·L^− 1^, which was 2.27-fold higher than that of the Landy 1 medium in shake flask fermentation. Meanwhile, the production of surfactin was scaled up to the 5 L fermenter level using foam reflux, and the yield of surfactin reached 2.39 g·L^− 1^ after 42.8 h of fermentation under optimal process conditions, which was 2.96-fold higher than that of the Landy 1 medium in 5 L fermenter. *B. subtilis* YPS-32 may represent a candidate strain for the industrial production of surfactin, and its optimized medium and culture conditions can be used as a reference for the industrial production of surfactin.

## Data Availability

All data generated or analysed during this study are included in this published article.

## Abbreviations

ARTP: atmospheric and room temperature plasma
CPC-BTB: cetylpyridinium chloride-bromothymol blue
TFA: Trifluoroacetic acid
HPLC: High Performance Liquid Chromatography
PBD: Plackett-Burman design
BBD: Box-Behnken design
